# Seasonal phenology of *Amauromyza karli* (Diptera: Agromyzidae) in quinoa in San Luis Valley of Colorado

**DOI:** 10.1093/ee/nvag069

**Published:** 2026-06-23

**Authors:** Neha Panwar, Lara M Amiri-Kazaz, Adrianna Szczepaniec

**Affiliations:** Department of Agricultural Biology, Colorado State University, Fort Collins, CO, USA; Department of Agricultural Biology, Colorado State University, Fort Collins, CO, USA; Department of Agricultural Biology, Colorado State University, Fort Collins, CO, USA

**Keywords:** *Chenopodium quinoa*, integrated pest management (IPM), pest monitoring, ordinal day model

## Abstract

*Amauromyza karli* Hendel (Diptera: Agromyzidae) has recently emerged as a damaging stem-boring pest of quinoa in Colorado and other quinoa-producing regions of the United States. Severe infestations have been associated with substantial yield losses, highlighting the need for improved understanding of the pest’s seasonal dynamics to support integrated pest management (IPM) strategies. The objective of this study was to characterize the seasonal phenology of *A. karli* in quinoa production systems in the San Luis Valley of Colorado. Adult flight activity was monitored using yellow sticky traps over 4 growing seasons, and larval incidence and stem exit holes were quantified across 2 years. Our results indicated that larval densities peaked in early June with infestations reaching over 90% by mid-July. An ordinal-day, 2-parameter logistic model based on cumulative trap captures was the most parsimonious model and predicted that 50% of adult activity occurred around late June. In addition, a negative binomial mixed-effects model showed no significant association between quinoa field size and adult captures, suggesting that variation in pest pressure is driven by field-level heterogeneity and temporal factors. These findings provide a framework for improving monitoring and management of *A. karli*. Aligning the timing of biological or chemical control measures with periods of peak adult activity, as well as adjusting planting dates to reduce exposure of vulnerable crop stages, may help mitigate damage caused by this pest. Overall, this study contributes foundational phenological information that can be used to inform IPM decision-making and reduce economic losses in quinoa production.

## Introduction

Quinoa, *Chenopodium quinoa* Willd (Caryophyllales: Ama­ranthaceae), is a pseudocereal grown worldwide for its nutrient-rich seeds and tolerance to abiotic stresses such as drought and salinity (Gesinski 2008, Bazile et al. 2016, Angeli et al. 2020, Asher et al. 2022, Yeşil and Levent 2022, Andreotti et al. 2023). Quinoa cultivation in the United States started in Colorado in 1982 (Goldberger and Detjens 2019), and since then the crop has expanded to other states such as Idaho (O’Connell 2015), Oregon (Buckland 2020), and Washington (Oeller et al. 2021). However, recent outbreaks of a stem-boring fly *Amauromyza karli* Hendel (Diptera: Agromyzidae) have caused severe yield losses in Colorado (Szczepaniec and Alnajjar 2023). The fly oviposits on quinoa stems and larvae develop within the pith, resulting in extensive plant injury. As a consequence of these infestations, quinoa acreage in Valley region of Colorado declined from approximately 1210 ha in 2021 to only 360 ha in 2022 (Szczepaniec and Alnajjar 2023). With an estimated yield potential of 1700 kg ha^−1^ and a bulk price of $6 per kg, the loss of 850 acres translates to an economic impact of approximately $8.5 million in lost quinoa production (Global Quinoa Price 2025).

This fly is native to the Palaearctic region and is considered an oligophagous species feeding primarily on *Chenopodium* spp. (EPPO Global database 2023). It has been recorded across parts of Europe, the Mediterranean region, and Asia; however, *A. karli* has not been reported as a key pest of quinoa in those regions. In North America, the fly was first documented in Ontario, Canada, in 1969. It was likely also present in the United States, but was not confirmed until 2021, when it was first reported from quinoa fields in Colorado (Szczepaniec and Alnajjar 2023). Little is known about the biology of this fly, and the only records of it were published by Boucher (2012) and Lonsdale (2021). To date, the oviposition behavior and egg morphology of *A. karli* remain undocumented. However, adults of related stem-boring agromyzids such as *Ophiomyia* spp. typically deposit eggs within young stems, petioles, or nodes (Ferro and Gilbertson 1982). Following hatching, the larvae of *A. karli* feed internally within quinoa stems and disrupt vascular transport which leads to reduced water and nutrient uptake (Szczepaniec and Alnajjar 2023). This physiological stress results in plant wilting and lodging, ultimately causing severe yield losses. Upon completing their development, larvae exit the stem and pupate in the soil. In stem-boring agromyzids, overwintering usually occurs in pupal stage with diapause primarily regulated by temperature and photoperiod. Short days and cool conditions can induce or sustain diapause in agromyzids, leading to synchronized spring emergence that coincides with host plant availability (Spencer 1973, Parrella 1987).

The limited understanding of the life cycle and seasonal activity of *A. karli* complicates the development of effective management strategies. Cultural practices such as crop rotation or adjusting planting dates could potentially reduce infestation pressure. However, without information on the timing of adult and larval activity, these approaches cannot be implemented strategically. Establishing the seasonal phenology of *A. karli* is therefore a critical first step toward identifying practical windows for management. Evidence from related systems supports this approach. For instance, in bean fly, *Ophiomyia phaseoli* Tryon (Diptera: Agromyzidae), adjusting sowing dates significantly reduced plant mortality from 43% in late sown peas to 12% in early sown crops suggesting that phenology-informed planting can lower stem fly injury (Yadav et al. 2018). Similarly, Kumar et al. (2024) reported that adjusting planting dates allowed sorghum to escape much of the injury caused by sorghum shoot fly, *Atherigona soccata* Rondani (Diptera: Muscidae). Deadheart incidence (tillers whose central whorl was killed and dried due to larval feeding) was reduced by nearly 50% in early-sown plots compared with late-sown sorghum. Furthermore, Buntin et al. (1990) demonstrated that delaying planting until early November reduced larval infestation by 90% compared to October plantings. Planting after the local “fly-free date” consistently avoided infestation by the damaging fall generation of Hessian fly, *Mayetiola destructor* Say (Diptera: Cecidomyiidae) in winter wheat (Buntin et al. 1990). These findings underscore the importance of aligning cultural practices with pest phenology to enhance control efficacy and reduce reliance on chemical inputs.

To address the lack of temporal data on *A. karli* activity, we used a phenology modeling approach to characterize its seasonal dynamics. Given the absence of detailed thermal biology of this pest, we used an ordinal-day model to predict adult fly activity. Ordinal models, which employ the day of the year to predict pest activities, provide a straightforward approach that can be particularly valuable for growers in the field (Davis and Hansen 2018). These models base their predictions on the calendar date, simplifying the monitoring and management of pests without the need for complex calculations or equipment. This simplicity not only makes the models more accessible to a broader range of users but also reduces the likelihood of errors that can occur with more complex phenological modeling approaches (Sridhar and Reddy 2013). By reducing complexity, these models help ensure that more growers can implement effective pest management strategies, ultimately leading to better crop protection and reduced losses due to pests (Davis and Hansen 2018). They can be particularly useful when species biology is poorly known because they require only calendar dates yet still summarize recurring seasonal patterns. Although their use in pest management is limited, they have performed well for insects with relatively predictable seasonal schedules (eg spruce beetle; Davis and Hansen 2018).

We therefore designed this study to understand seasonal phenology of *A. karli.* Specifically, we (i) quantified adult flight activity across sites and years, (ii) tracked larval incidence and exit holes throughout the growing season, and (iii) developed an ordinal-day model to predict the onset, peak, and decline of adult activity. The outcomes of this work will translate seasonal observations into actionable management tactics. Identifying specific activity periods of *A. karli* will allow for precise timing required to deploy monitoring traps and target the susceptible life stages. Additionally, these data facilitate the development of cultural strategies such as adjusting planting dates and crop rotation to minimize infestation pressure.

## Materials and Methods

### Monitoring of Adult *A. karli*

Field surveys were conducted in grower fields in the San Luis Valley region of Colorado over 4 growing seasons, from 2022 to 2025 (Supplementary Table S1). The San Luis Valley in south-central Colorado has been a key region for quinoa cultivation in North America since the 1980s because its 2300 m elevation and arid climate closely resemble the crop’s native Andean conditions. There were a total of 12 site × year combinations with 4 fields sampled in 2022, 3 fields surveyed in 2023, 3 fields surveyed in 2024, and 2 fields sampled in 2025. Regional quinoa acreage declined substantially over the study period, reducing the number of available sampling sites each year. This decline was largely attributed to *A. karli* infestations, which growers cited as a key factor in reducing quinoa cultivation.

**Fig. 1.**
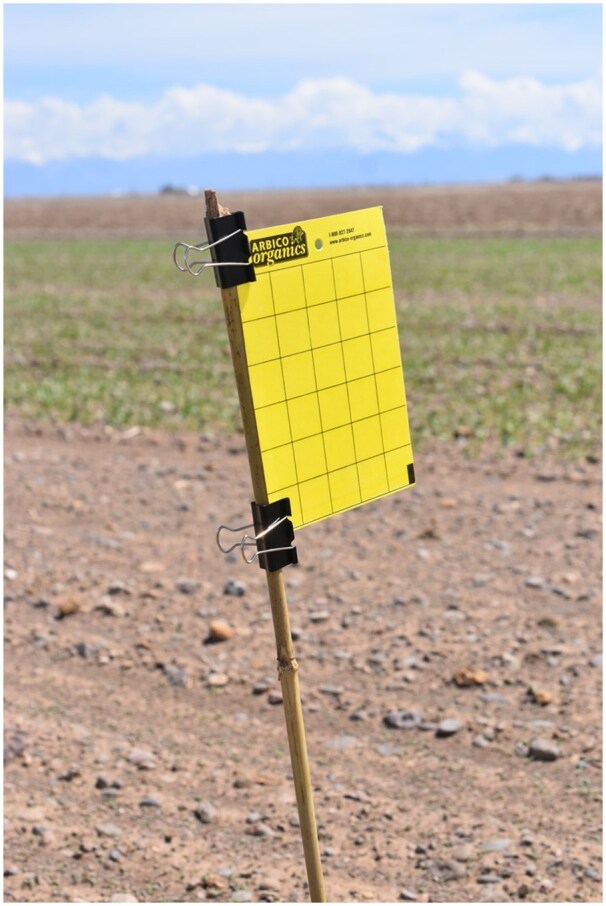
Yellow sticky trap setup used to monitor adult *Amauromyza karli* in quinoa fields. A 12.7 × 17.78 cm sticky card (ARBICO Organics Yellow Insect Traps) was affixed to a bamboo stake using metal binder clips. Stakes were initially 30 cm long and were adjusted during the season to maintain trap placement at canopy height.

To monitor the adult fly activity, yellow sticky traps measuring 12.7 cm × 17.78 cm (ARBICO Organics Yellow Insect Traps) were affixed to bamboo sticks using a metal clip (Fig. 1). Traps were placed at least 100 m apart within each field. Traps were initially placed at a height of around 30 cm and were then adjusted as needed to maintain traps at canopy height. We used yellow sticky traps because adult agromyzids are highly attracted to yellow color (Martin et al. 2005, Hamza et al. 2023). The traps were deployed within 15 days of sowing and their number in each field and trap placement dates varied across the years (Supplementary Table S1). In 2022, 8 yellow sticky traps were deployed in each field beginning on 6 June and replaced at weekly intervals until 10 August. In 2023, 6 traps were installed in each field beginning between 24 May and 13 June, depending on sowing dates, and were replaced weekly until 14 August. In 2024, 5 traps were deployed per field, with the first trap installed on 28 April in the early‑sown field and on 11 June in the late‑sown field; all traps were replaced weekly until 13 August. In 2025, 5 traps per field were installed beginning on 10 June, and traps were replaced at biweekly intervals until 23 July. The duration of trap deployment in the field was recorded for each trap.

**Fig. 2.**
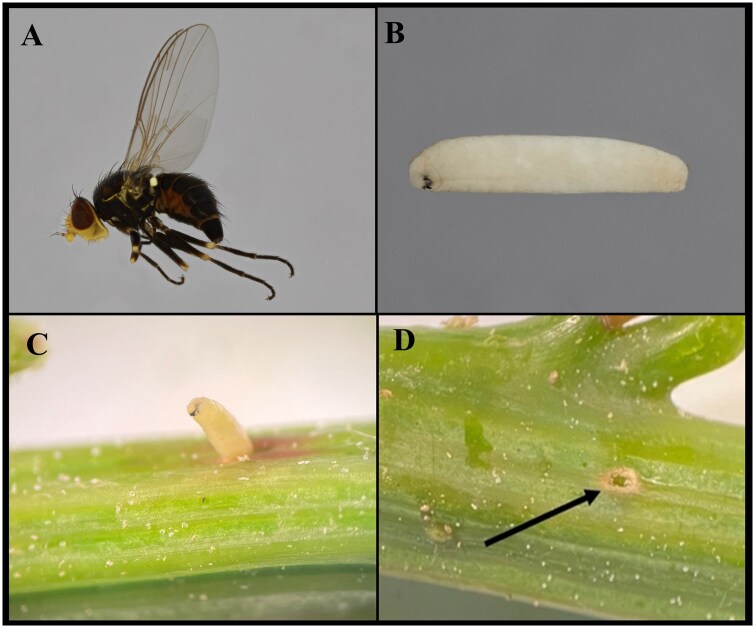
Representative images illustrating the diagnostic characters of *Amauromyza karli*. A) Adult *A. karli* is characterized by distinct coloration of the head, legs, and joints between leg segments, B) larvae of *A. karli* from quinoa stems in Colorado, C) a fully grown larva exiting the stems, and D) exit hole on the stem (arrow) created by larva of *A. karli*. Reproduced from Szczepaniec and Alnajjar (2023) under the Creative Commons Attribution-NonCommercial (CC BY-NC 4.0) license.

Upon collection from the field, traps were covered with parafilm to separate the traps from each other, and transported in coolers with ice bags to the laboratory at Colorado State University and refrigerated at 4 °C. The traps were thoroughly inspected using a dissecting microscope (Jenco DG6-2L 7-45X Stereo Zoom Binocular Microscope, Jenco International, Portland, OR 97222) to assess the number of adult *A. karli*. The adults were identified using their characteristic yellow head and yellow joints between femur and tibia (Lonsdale 2021; Fig. 2). The traps were processed within a month after they arrived in the laboratory. Fly captures at each location were transformed into cumulative captures (percentage of total), resulting in a trap capture accumulation curve for each site and year combination.

### Sampling for *A. karli* Larvae

Larval and exit hole densities were quantified in 2024 and 2025 in the same fields where adults were sampled. We counted live larvae within the stems and simultaneously recorded exit holes on the stem surface. Exit holes are created by mature larvae of *A. karli* when they exit the quinoa stems to pupate in the soil. These exit holes served as a proxy for the larvae that had completed their development prior to our sampling.

In 2024, 50 plants per field were destructively sampled at biweekly intervals, while in 2025, 30 plants were sampled per field. Sampling began on 4 June in both years and ended on 13 August in 2024 and 23 July in 2025. Field sizes ranged from 0.2 ha to 64 ha in area. At each location, 5 sampling sections were established that were separated by at least 100 m. Within each section, 10 plants spaced at least 5 m apart were sampled in 2024, and 6 plants separated by at least 5 m were randomly selected from each section in 2025 (*N* = 50 in 2024 and *N* = 30 in 2025 for each field and each sampling date). Plants were clipped at the base with pruners, placed in labeled bags, transported on ice to the laboratory at Colorado State University, kept at room temperature and processed within 24 h. Stems were split longitudinally with a scalpel, and the number of larvae and exit holes per stem were recorded along with stem length and diameter. Stem diameter was measured using a vernier caliper, and larval and exit hole densities were expressed per cm³ of stem tissue.

### Ordinal Day Model Construction

Sigmoid functions are frequently used to model cumulative trap capture data as they represent the gradual accumulation of insects over time (Jones et al. 2016, Davis and Hansen 2018). We evaluated 3 different 2-parameter sigmoid models to predict the cumulative captures: logistic, Gompertz, and Weibull curves. In all ordinal-day models, day of the year was used as a predictor variable for the ordinal day model, and field and year were included as random effects. All models were constructed using nlme function in R to predict cumulative trap capture data (Elzhov et al. 2022). We compared the models using Akaike’s information criterion (AIC) and selected the model with lowest AIC as the most parsimonious for describing cumulative trap captures. All analyses were performed in R (R Core Team 2025).

### Association Between Adult Abundance and Quinoa Acreage

A generalized linear mixed effects model (GLMM) with negative binomial distribution was fitted in R using the glmmTMB function (Brooks et al. 2017) to evaluate the relationship between *A. karli* counts and quinoa acreage. Log-transformed field area (ha) was included as a fixed effect. Random intercepts were included for year, day of year, field ID, and trap ID to account for temporal variation and repeated measurements. Trap replacement interval (number of days) was included as an offset term to account for variation in trap deployment duration among sampling intervals. All analyses were performed in R (R Core Team 2025).

## Results

### Seasonal Activity of *A. karli* Adults and Larvae

**Fig. 3.**
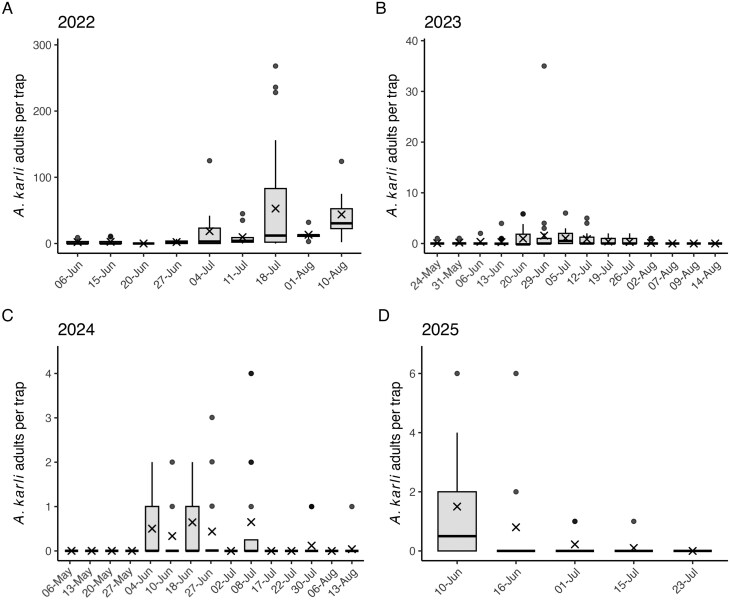
Box plots showing the seasonal activity of *Amauromyza karli* adults across survey years. Data were pooled across all locations within each year to illustrate generalized trends in adult fly activity in 2022 (A), 2023 (B), 2024 (C), and 2025 (D). The *x*-axis represents sampling dates, while the *y*-axis indicates the number of *A. karli* adults per trap. Boxes represent the interquartile range (25th to 75th percentiles) of the numbers of *A. karli* adults per trap across time with the median indicated by the lines across bars, while “x” marks the mean. Black markers denote outliers. The seasonal activity of *A. karli* varied across years. In 2022, maximum fly numbers for a single trap were 250 (A), whereas in 2023 it decreased to 35 (B), and to only 4 and 6 in 2024 (C) and 2025 (D) respectively.

Adults of *A. karli* were active in quinoa fields from late May through mid-August (Fig. 3). Over the course of 4 years, 2,752 adults of *A. karli* were caught on the yellow sticky traps. On average, 688 ± 599.05 adult flies were captured each year, and ranged from 26 to 2,481 adults. The highest number of individuals, ie 2,481 adults were recorded in 2022 whereas the lowest number of 26 individuals were captured in 2025.

The timing of adult *A. karli* captures varied across years. In both 2022 and 2023, adult *A. karli* were recovered on the first sampling dates (6 June and 17 May, respectively; Fig. 3), indicating that flight activity had commenced prior to trap deployment. Peak captures occurred on 18 July in 2022 and on 20 June in 2023. By contrast, the earlier initiation of trapping in 2024 enabled clearer resolution of emergence timing: traps deployed in late April yielded no detections until 27 May, delineating the onset of activity in quinoa fields. In 2025, population density was markedly reduced, and adults were detected only intermittently between 10 June and 26 July. Likewise, peak trap captures also differed over the study period. The highest activity was recorded in 2022 (maximum of 250 flies per trap), followed by a reduction in 2023 (35 flies per trap). In 2024 and 2025, maximum captures of only 4 and 6 flies per trap were reported respectively.

**Fig. 4.**
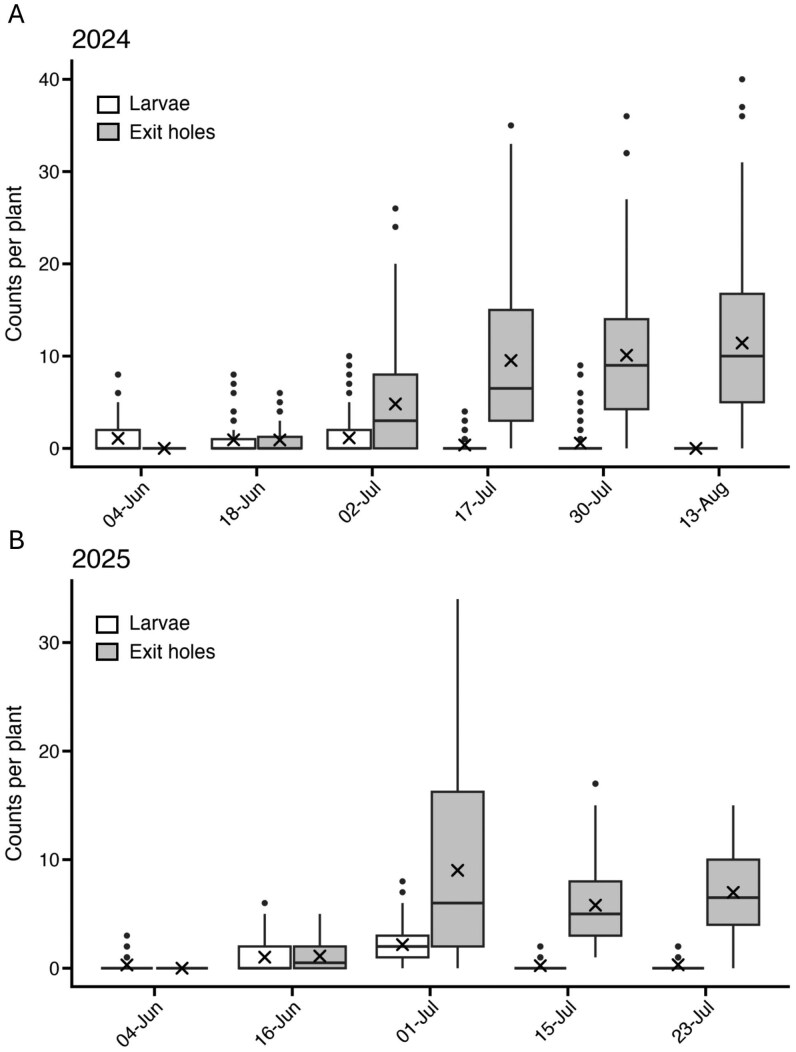
Seasonal progression of *Amauromyza karli* larval infestation and exit hole formation. Boxplots show the distribution of larvae and exit hole counts per plant across sampling dates in 2024 (A) and 2025 (B), with larvae represented in white and exit holes in gray. Boxes represent the interquartile range (25th to 75th percentiles) with the median indicated by the lines across bars, while “x” marks the mean. Black markers denote outliers. These patterns illustrate within‑season changes in larval abundance and the timing of larval development and emergence.

Across both years, larval activity began in early June, peaked in early July, and declined sharply by mid-July (Fig. 4). In 2024, larval densities increased from 1.09 ± 0.17 larvae per plant on 4 June to a peak of 1.14 ± 0.17 on 2 July. In 2025, larvae followed a similar pattern but reached a higher peak of 2.17 ± 0.24 on 1 July. By mid-July in both years, larval densities began to decline, with no larvae detected by mid-August in 2024 and only 0.32 ± 0.08 larvae per plant by late July in 2025.

**Fig. 5.**
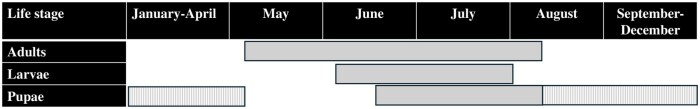
Estimated phenological timeline of *Amauromyza karli* life stages in quinoa fields in Colorado. The figure summarizes field observations from adult trap captures, larval counts, and exit holes, indicating periods of adult activity, larval feeding, pupation, and overwintering. Adult activity begins in early May, likely emerging from overwintering pupae, and continues through early August. Larval feeding is first observed in early June, followed by the appearance of initial exit holes in late June. Hatched bars denote overwintering pupae.

Exit holes were absent during early June sampling in both years (Fig. 4), suggesting that larvae present at that time represented the first generation (Fig. 5). Exit holes first appeared in mid-June in both the years and increased steadily until mid-August. In 2024, exit holes increased from 0.91 ± 0.14 per plant on 18 June to 11.4 ± 0.69 by 13 August. In 2025, exit hole densities peaked at 9.02 ± 1.04 per plant on 1 July and remained high until late July (Fig. 4). Infestation levels increased from 41% to 96% by July end in 2024 whereas in 2025, all the plants were infested by mid-July.

Our field observations of adult and larval activity suggest a distinct seasonal progression of *A. karli* life stages in Colorado quinoa fields (Fig. 5). Overwintering likely occurred in the pupal stage from approximately September through April. Adult activity began in early May and continued through early August, suggesting emergence from overwintering pupae in late spring. Larval feeding within stems was first detected in early June and persisted through mid- to late July. The first exit holes, indicating completion of larval development and transition to pupation, were observed in late June, and the continued larval activity and exit holes in July suggest at least 2 generations per season. Pupation occurred during mid-summer and was followed by the overwintering pupal stage that persisted through fall, winter, and early spring (Fig. 5).

### Ordinal Day Model

**Fig. 6.**
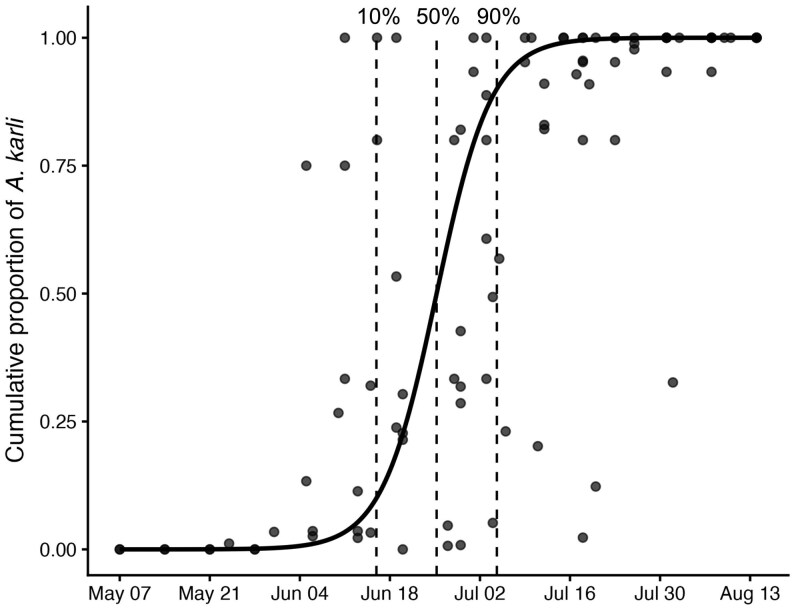
Seasonal phenology of *Amauromyza karli* based on a mixed-effects logistic model. The graph depicts the cumulative proportion of *A. karli* emergence as a function of the day of the year. Open circles represent observed data points. The solid black line represents the fitted logistic model. The *x*-axis denotes the day of the year, and the *y*-axis represents the cumulative proportion of *A. karli* emergence. The ordinal day model estimates that 10%, 50%, and 90% of *A. karli* trap captures occur on 16 June, 26 June, and 5 July, respectively. These dates mark the approximate initiation, peak, and cessation of fly activity. The sigmoidal pattern of emergence highlights a gradual increase in adult fly captures, followed by a peak and a plateau.

We evaluated 3 different 2-parameter logistic functions (Gompertz, logistic, and Weibull) using ordinal day to predict fly activity. The 2-parameter logistic model was the most parsimonious with the lowest AIC (Supplementary Fig. S1). The logistic ordinal-day model can be used to predict cumulative fly activity using only day of year as input, using the following equation:


$$\begin{array}{l} \text{Percentage of }A.\;karli\text{ trap capture}\\ =\frac{1}{1+exp\;\left(-\frac{ordinal\;day-I}{S}\right)} \end{array}$$

where *I* is the inflection point which denotes the day at which 50% of cumulative fly capture occurs, and *S* is the scale parameter controlling curve steepness. At the population-level (fixed-effects model), the estimated inflection point was 176.26 ± 7.31 (*t* *=* 24.10; *P *< 0.001; Table 1), indicating that 50% of cumulative fly capture occurred by day 176 (26 June). The steepness parameter was 4.26 ± 0.37 (*t* *=* 11.26; *P *< 0.001). Random effects showed variation among years and among sites nested within year, reflecting spatial and temporal heterogeneity in emergence timing. This model explained 95.6% of the variance in cumulative *A. karli* captures (RMSE = 0.956). The ordinal day model predicted 10%, 50%, and 90% of the cumulative fly capture occurs on 16 June, 26 June, and 5 July respectively (Fig. 6). These trap capture dates can be used as an approximation for initiation of fly activity, peak activity and cessation of fly activity, respectively.

**Table 1.** Parameter estimates for the mixed-effects logistic ordinal-day model predicting cumulative proportion trap capture of *A. karli*

Effect type	Parameter	Estimate	Standard error	t-value	*P*-value
**Fixed effects**	Inflection point, *I*	176.26	7.31	24.10	<0.001
	Slope, S	4.26	0.38	11.26	<0.001
**Random effects**	Parameter	Variance	Standard deviation		
	Year	157.47	12.545		
	Site	141.56	11.90		
	Residual	0.008	0.09		

Fixed effects describe the population-level inflection point (*I*) and scale (*S*). Random effects were applied to *I* for year and site within year.

### Relationship Between Adult Abundance and Quinoa Acreage

**Fig. 7.**
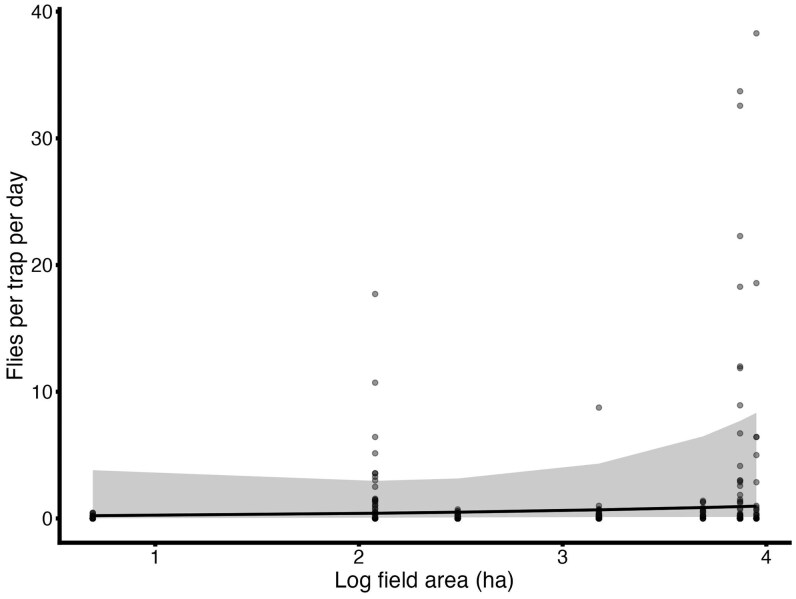
Relationship between quinoa field area and *Amauromyza karli* trap catch. Points show observed trap-level counts standardized by deployment duration (flies per trap-day). The solid line and shaded band show the model-predicted mean and 95% confidence interval from a negative binomial GLMM with log field area as a fixed effect, an offset for log days deployed, and random intercepts for year, field (site), day-of-year, and trap identity.

A negative binomial mixed‑effects model showed no significant association between quinoa field size and *A. karli* adult captures (Table 2, Fig. 7). The estimated effect of field area was positive but non-significant (*β* = 0.459 ± 0.527; *z* = 0.871; *P *= 0.384), suggesting that variation in trap catch was driven primarily by temporal and field-level heterogeneity rather than field size alone. Random effects for day of year, year, site, and trap ID accounted for substantial variation in trap counts, with day of year contributing the largest variance component (Table 2).

**Table 2.** Negative binomial generalized linear mixed model (GLMM) evaluating the relationship between *Amauromyza karli* trap counts and quinoa field area

Effect type	Parameter	Estimate	Standard error	z-value	*P*-value
**Fixed effects**	Intercept	−3.82	1.76	−2.174	0.028
	Area	0.46	0.53	0.871	0.384
**Random effects**	Parameter	Variance	Standard deviation		
	Day of year	3.07	1.75		
	Year	1.86	1.36		
	Site	1.65	1.28		
	Trap ID	0.11	0.33		

Field area was log-transformed. Random intercepts were included for day of year, year, site, and trap ID. Trap exposure duration was included as an offset term.

## Discussion

The objective of this study was to characterize the seasonal phenology of *A. karli* in quinoa production systems in Colorado and translate these patterns into recommendations that can inform integrated pest management (IPM) strategies. Using multi-year field monitoring of adult flight activity and larval development, we show that *A. karli* exhibits distinct seasonal peaks, with maximum adult activity occurring in late June and highest larval activity in early July. An ordinal-day model based on cumulative adult trap captures provided a simple and practical framework for estimating the timing of adult activity using only calendar date. Together, these results establish a temporal foundation for improving monitoring efficiency and for aligning management actions, such as the timing of scouting, planting decisions, and intervention measures, with periods of elevated pest activity. While these findings do not directly test management efficacy, they provide critical phenological context needed to develop and refine IPM approaches for this emerging pest of quinoa.

The 2-parameter ordinal day model is straightforward for growers to use as it requires only day of the year as an input to predict the cumulative fly captures (Sridhar and Reddy 2013). Similar ordinal day models have been used successfully in other systems. For example, one was applied to time trap placement for spruce beetle, improving monitoring efficiency (Davis and Hansen 2018). Our ordinal day model indicates that fly activity begins in mid-June, peaks in late June, and declines by early July. Based on this pattern, deploying traps in early June until late June would allow for effective monitoring of fly activity (Jones et al. 2016, Davis and Hansen 2018).

These phenological patterns also provide guidance for management timing. Aligning interventions with peak pest abundance can improve the effectiveness of control tactics. Because *A. karli* activity peaks in late June, management actions would likely be most effective if implemented shortly before or during this period. Conventional insecticides have shown limited effectiveness against this pest, and predators or parasitoids capable of reliably suppressing *A. karli* have not yet been identified. However, entomopathogenic fungi with endophytic potential such as *Beauveria bassiana* and *Metarhizium anisopliae* may provide a promising alternative for pests with concealed feeding habits (Akutse et al. 2013, Panwar and Szczepaniec 2024). These fungi could be applied either as seed treatment or as foliar application before the peak activity in late June to suppress the *A. karli* populations.

Given these phenological patterns, management tactics that disrupt the phenological synchrony of *A. karli* with the crop may be highly effective. For example, adjusting planting dates would reduce overlap between vulnerable crop stages and peak pest activity. Planting early in the season, such as late April, may allow quinoa plants to establish beyond the most vulnerable growth stages before peak *A. karli* activity begins in late June. Alternatively, delayed planting in early July could reduce early-season exposure to high fly populations, potentially minimizing infestation rates. However, late sowing has been shown to reduce quinoa growth and yield, particularly in arid climates. Taaime et al. (2022) reported that late planting led to stunted plants and lower grain yields as a result of the shortened growing season and heat stress during flowering. Thus, while shifting planting dates may help manage *A. karli*, this strategy must be carefully balanced with local climatic conditions to optimize pest control without compromising yield.

Our ordinal-day model offers a great tool to interpret adult *A. karli* captures and guide planting decisions; however, its application should be approached with caution as it does not encompass all the lifecycle stages of *A. karli*, specifically the larval stage. Furthermore, this model does not account for environmental conditions which may influence insect activity and behavior (Davis and Hansen 2018). For more precise modeling and prediction, site-specific weather data would be essential. Microclimates within specific fields can differ due to various factors like elevation, soil type, and other localized factors. Therefore, the predictive ability of these models can further be enhanced by incorporating factors which influence pest development directly or indirectly such as precipitation, relative humidity, soil moisture, and soil temperature (Olatinwo and Hoogenboom 2014). Also, development metrics such as thermal thresholds for *A. karli* are currently unknown. Future research should focus on estimating the lower/upper thresholds and thermal constants for different life cycle stages.

We observed considerable annual variation in fly densities, with a strong declining trend across survey years. Although both regional quinoa acreage and maximum fly density declined during the study period, our mixed-effects model showed no association between quinoa field size and adult fly captures. Since our study sites comprised grower fields distributed across the valley rather than a single continuous block of quinoa, this suggests that factors other than field size alone, including site-specific conditions might have played a stronger role in influencing adult densities. A major caveat of this study was that our 2022 sampling sites did not represent the total quinoa acreage in the region, potentially reducing our ability to detect relationships between host availability and adult fly abundance. We also observed greater *A. karli* pressure in fields that were continuously planted with quinoa compared to fields without quinoa during the previous season. This suggests that fly populations can be reduced not only by rotating individual fields out of quinoa, but also by increasing the distance between new quinoa fields and previous year’s heavily infested sites. A similar distance-based strategy has been used to suppress wheat stem sawfly, *Cephus cinctus* Norton (Hymenoptera: Cephidae), a stem-boring pest that overwinters in the previous year’s crop residue. Maximizing the isolation distance between new wheat plantings and infested stubble significantly limits dispersal and reduces infestation severity (Beres et al. 2011).

Determining the overwintering stage of *A. karli* is crucial for developing targeted management practices. Field observations indicated that *A. karli* completes its larval development within quinoa stems, with mature larvae exiting stems in mid to late August and pupating in the soil near the root zone. Although overwintering pupae were not directly recovered, the available biological evidence strongly indicates that *A. karli* overwinters in the pupal stage. Alternative hypotheses are constrained by the life-history traits of the Agromyzidae. First, overwintering as soil-deposited eggs is highly improbable because agromyzid females are obligate endophytes that deposit eggs exclusively within plant tissue rather than into the soil (Spencer 1973). Second, the consistent presence of larval exit holes confirms that mature larvae exit the stem. In the Agromyzidae, larvae typically pupate immediately after exiting the host substrate. Finally, both larval and adult activity ended by August in all years of sampling, with no late season captures. This pattern also aligns with other agromyzid stem borers, such as *O. phaseoli* and *Melanagromyza sojae* Zehntner (Diptera: Agromyzidae), which pupate in soil and rely on temperature and photoperiod cues to regulate diapause and spring emergence (Spencer 1973). Together, this suggests that soil-dwelling puparium is the most parsimonious and biologically consistent overwintering stage. We did not conduct thorough, targeted soil searches for overwintering puparia, however. Future studies should prioritize the direct recovery of overwintering puparia, either through targeted soil sampling in late fall and early spring or through spring emergence trapping, to confirm the overwintering stage of *A. karli.*

Likely overwintering habits of *A. karli* suggest that management strategies could include disrupting the soil-dwelling pupal stage. After harvesting, cultural practices such as soil disturbance or shallow tillage could help expose overwintering pupae to desiccation, temperature extremes, or predation reducing carryover populations to the following season. Similar tactics have been recommended for other insects which pupate in soil. For example, shallow tillage during fall can cause up to 90% mortality of wheat stem sawfly which pupates in wheat stubbles (Government of Manitoba 2025). However, in the San Luis Valley, shallow tillage may lead to increasing the risk of wind erosion and topsoil loss. Also, disturbing the soil profile in autumn may reduce winter snow capture, which is an important source of aquifer recharge in this arid region. Therefore, implementation should prioritize partial disturbance methods such as strip tillage to mitigate soil loss in this region.

In addition to cultural practices, the soil-dwelling pupal stage can be targeted for biological control. Since *A. karli* larvae exit the stem to pupate in the soil, they can become vulnerable to soil-applied entomopathogens. This approach has proven effective against other agromyzid leaf miners. For example, Mugala et al. (2022) showed that pupae of pea leafminer*, Liriomyza huidobrensis* Blanchard (Diptera: Agromyzidae), were highly susceptible to soil-applied biocontrol agents, with 57.3% mortality caused by entomopathogenic nematode *Heterorhabditis baujardi* and 81.4% mortality when treated with the fungus *Metarhizium robertsii*. Soil applications of *M*. *anisopliae* under field-cage conditions have also suppressed pupal stages of the Mexican fruit fly, *Anastrepha ludens* Loew (Diptera: Tephritidae), by reducing adult emergence from 76% to 33% in loam soil and from 71% to 49% in sandy loam soil (Lezama-Gutiérrez et al. 2000). Furthermore, granular formulations of *M. anisopliae* have demonstrated long-term persistence in the soil profile. For example, Ekesi et al. (2005) found that a single application of *M. anisopliae* continued to suppress adult emergence of Mediterranean fruit fly, C*. capitata* Wiedemann (Diptera: Tephritidae) by 37% even 668 days after treatment. These results suggest that timed soil applications of entomopathogenic fungi or nematodes can be explored as a tactic to suppress *A. karli* in quinoa fields.

In conclusion, our study represents an initial step toward understanding the seasonal phenology of *A. karli.* This information can improve *A. karli* monitoring and management in quinoa fields. The ordinal day model offers a practical and effective approach for growers in Colorado. Future work should focus on expanding this model across different geographic areas and incorporating additional environmental variables to further improve their predictive power. As climate variability continues to impact agricultural systems, developing accurate and region-specific phenology models will be crucial for enhancing IPM strategies and ensuring sustainable quinoa production.

## Supplementary Material

nvag069_Supplementary_Data

## References

[nvag069-B1] Akutse K , Maniania N, Fiaboe K, et al 2013. Endophytic colonization of *Vicia faba* and *Phaseolus vulgaris* (Fabaceae) by fungal pathogens and their effects on the life-history parameters of *Liriomyza huidobrensis* (Diptera: Agromyzidae). Fungal Ecol. 6:293–301. 10.1016/j.funeco.2013.01.003

[nvag069-B2] Andreotti F , Neher CM, Speelman EN, et al 2023. Exploring farmers’ perspectives on agrobiodiversity management: future options for quinoa smallholder organizations in the Peruvian high Andes. Agron. Sustain. Dev. 43:1–15. 10.1007/s13593-023-00891-y

[nvag069-B3] Angeli V , Miguel Silva P, Crispim Massuela D, et al 2020. Quinoa (*Chenopodium quinoa* Willd.): an overview of the potentials of the “Golden Grain” and socio-economic and environmental aspects of its cultivation and marketization. Foods. 9:1–31. 10.3390/foods9020216 PMC7074363 32092899

[nvag069-B4] Asher A , Dagan R, Galili S, et al 2022. Effect of row spacing on quinoa (*Chenopodium quinoa*) growth, yield, and grain quality under a mediterranean climate. Agriculture. 12:1298. 10.3390/agriculture12091298

[nvag069-B5] Bazile D , Pulvento C, Verniau A, et al 2016. Worldwide evaluations of quinoa: preliminary results from post international year of quinoa FAO projects in nine countries. Front. Plant Sci. 7:1–18. 10.3389/fpls.2016.00850 27446101 PMC4914551

[nvag069-B6] Beres BL , Dosdall LM, Weaver DK, et al 2011. Biology and integrated management of wheat stem sawfly and the need for continuing research. Can. Entomol. 143:105–125. 10.4039/n10-056

[nvag069-B7] Boucher S. 2012. Revision of the Canadian species of *Amauromyza* Hendel (Diptera: Agromyzidae). Can. Entomol. 144:809–833. 10.4039/tce.2012.80

[nvag069-B8] Brooks ME , Kristensen K, van Benthem KJ, et al 2017. glmmTMB balances speed and flexibility among packages for zero-inflated generalized linear mixed modeling. R. J. 9:378–400. 10.32614/RJ-2017-066

[nvag069-B9] Buckland K. 2020. Quinoa production for the Willamette Valley. Oregon State University Extension Publication EM 9300. https://extension.oregonstate.edu/catalog/em-9300-quinoa-production-willamette-valley

[nvag069-B10] Buntin DG , Bruckner PL, Johnson JW. 1990. Management of hessian fly (Diptera: Cecidomyiidae) in Georgia by delayed planting of winter wheat. J. Econ. Entomol. 83:1025–1033. 10.1093/jee/83.3.1025

[nvag069-B11] Davis TS , Hansen EM. 2018. An ordinal day model of spruce beetle trap capture phenology in northern Colorado. J. Appl. Entomol. 142:277–281. 10.1111/jen.12424

[nvag069-B12] Ekesi S , Maniania NK, Mohamed SA, et al 2005. Effect of soil application of different formulations of *Metarhizium anisopliae* on African tephritid fruit flies and their associated endoparasitoids. Biol. Control. 35:83–91. 10.1016/j.biocontrol.2005.06.010

[nvag069-B13] Elzhov TV , Mullen KM, Spiess AN, et al 2022. minpack.lm: R Interface to the Levenberg-Marquardt nonlinear least-squares algorithm found in MINPACK, Plus Support for Bounds. R package version 1.2–4. 10.32614/CRAN.package.minpack.lm

[nvag069-B14] EPPO Global database. 2023. *Amauromyza karli:* an emerging pest of quinoa in the USA [accessed 10 February 2026]. https://gd.eppo.int/reporting/article-7566

[nvag069-B15] Ferro DN , Gilbertson RL. 1982. Bionomics and population dynamics of the asparagus miner, *Ophiomyia simplex* (Loew), in western Massachusetts 1. Environ. Entomol. 11:639–644. 10.1093/ee/11.3.639

[nvag069-B16] Gesinski K. 2008. Evaluation of the development and yielding potential of *Chenopodium quinoa* Willd. under the climatic conditions of Europe. Part two: yielding potential of *Chenopodium quinoa* under different conditions. Acta Agrobot. 61:185–189. 10.5586/aa.2008.026

[nvag069-B17] Global Quinoa Price. 2025. Tridge [accessed 4 November 2025]. https://dir.tridge.com/prices/quinoa

[nvag069-B18] Goldberger J , Detjens A. 2019. Organic farmers’ interest in quinoa production in the western United States. Food Stud. 9:17–35. 10.18848/2160-1933/CGP/v09i03/17-35

[nvag069-B19] Government of Manitoba. 2025. Wheat stem sawfly. Government of Manitoba: agriculture [accessed 7 November 2025]. https://www.gov.mb.ca/agriculture/crops/insects/wheat-stem-sawfly.html

[nvag069-B20] Hamza MA , Ishtiaq M, Mehmood MA, et al 2023. Management of vegetable leaf miner, *Liriomyza* Spp., (Diptera: Agromyzidae) in vegetable crops. Horticulturae. 9:255–212. 10.3390/horticulturae9020255

[nvag069-B21] Jones VP , Horton DR, Mills NJ, et al 2016. Using plant volatile traps to develop phenology models for natural enemies: an example using *Chrysopa nigricornis* (Burmeister) (Neuroptera: Chrysopidae). Biol. Control. 102:77–84. 10.1016/j.biocontrol.2014.12.012

[nvag069-B22] Kumar R , Kumar D, Sharma BL, et al 2024. Impact of date of sowing on the incidence of sorghum stem borer, *Chilo partellus* and sorghum shoot fly, *Atherigona soccata* in dual purpose sorghum. Forage Res. 49:486–492.

[nvag069-B23] Lezama-Gutiérrez R , La Luz AT, Molina-Ochoa J, et al 2000. Virulence of *Metarhizium anisopliae (*Deuteromycotina: Hyphomycetes) on *Anastrepha ludens* (Diptera: Tephritidae): laboratory and field trials. J. Econ. Entomol. 93:1080–1084. 10.1603/0022-0493-93.4.1080 10985015

[nvag069-B24] Lonsdale O. 2021. Manual of North American Agromyzidae (Diptera, Schizophora), with revision of the fauna of the “Delmarva” states. Zookeys. 1051:1–481. 10.3897/zookeys.1051.64603 34393548 PMC8342412

[nvag069-B25] Martin AD , Vernon RS, Hallett RH. 2005. Influence of colour and trap height on captures of adult pea leafminer, *Liriomyza huidobrensis* (Blanchard) (Diptera: Agromyzidae), in celery. J. Ent. Soc. Ont. 136:25–35.

[nvag069-B26] Mugala T , Visser D, Malan AP, et al 2022. Entomopathogens from agricultural soil and their pathogenicity against the potato leaf miner, *Liriomyza huidobrensis* (Diptera: Agromyzidae). Biocontrol. Sci. Technol. 32:952–970. 10.1080/09583157.2022.2070128

[nvag069-B27] O’Connell J. 2015. Eastern Idaho reports strong quinoa yields. Capital Press. https://capitalpress.com/2015/08/25/eastern-idaho-reports-strong-quinoa-yields/

[nvag069-B28] Oeller E , Clark R, Hinojosa L, et al 2021. Effects of agronomic practices on *Lygus* spp. (Hemiptera: Miridae) population dynamics in quinoa. Environ. Entomol. 50:852–859. 10.1093/ee/nvab039 33960388

[nvag069-B29] Olatinwo R , Hoogenboom G. 2014. Weather-based pest forecasting for efficient crop protection. In: Abrol DP, editor. Integrated pest management, current concepts and ecological perspective. Elsevier. p. 59–78. 10.1016/B978-0-12-398529-3.00005-1

[nvag069-B30] Panwar N , Szczepaniec A. 2024. Endophytic entomopathogenic fungi as biological control agents of insect pests. Pest Manag. Sci. 80:6033–6040. 10.1002/ps.8322 39046187

[nvag069-B31] Parrella MP. 1987. Biology of *Liriomyza*. Annu. Rev. Entomol. 32:201–224. 10.1146/annurev.en.32.010187.001221

[nvag069-B32] R Core Team. 2025. R: A language and environment for statistical computing. 4.4.3.Vienna. R Foundation for Statistical Computing.

[nvag069-B33] Spencer KA. 1973. Agromyzidae (Diptera) of economic importance. Springer Netherlands. 10.1007/978-94-017-0683-4

[nvag069-B34] Sridhar V , Reddy PVR. 2013. Use of degree days and plant phenology: a reliable tool for predicting insect pest activity under climate change conditions. In: Singh HCP, Rao NKS, Shivashankara KS, editors. Climate-resilient horticulture: adaptation and mitigation strategies. Springer. p. 287–294. 10.1007/978-81-322-0974-4_25

[nvag069-B35] Szczepaniec A , Alnajjar G. 2023. New stem boring pest of quinoa in the United States. J. Integr. Pest Manag. 14:1–7. 10.1093/jipm/pmad004

[nvag069-B36] Taaime N , Mejahed KE, Moussafir M, et al 2022. Early sowing of quinoa cultivars, benefits from rainy season and enhances quinoa development, growth, and yield under arid condition in Morocco. Sustainability. 14:4010–4019. 10.3390/su14074010

[nvag069-B37] Yadav A , Singh V, Yadav A, et al 2018. Effect of dates of sowing on the incidence of pea stemfly, *Ophiomyia phaseoli* (Tryon) on pea In Rajasthan. Bull. Env. Pharmacol. Life Sci. 7:71–74. 10.13140/RG.2.2.15541.09443

[nvag069-B38] Yeşil S , Levent H. 2022. The influence of fermented buckwheat, quinoa and amaranth flour on gluten-free bread quality. LWT Food Sci. Technol. 160:113301–113308. 10.1016/j.lwt.2022.113301

