# Supplementary material for: Seasonal phenology of Amauromyza karli (Diptera: Agromyzidae) in quinoa in San Luis Valley of Colorado
Source: Environ Entomol. 2026 Jun 23;55(3):nvag069. doi: 10.1093/ee/nvag069 (PMC13287995; doi:10.1093/ee/nvag069)

Supplementary Figure S1. Observed versus predicted cumulative proportion of seasonal *Amauromyza karli* trap captures. Three different ordinal-day phenology models were evaluated: two -parameter Gompertz, two-parameter logistic, and two-parameter Weibull, each fitted as nonlinear mixed-effects models. Points represent site-year observations of cumulative proportion emergence and each panel shows model fit relative to a 1:1 reference line. Akaike’s Information Criterion (AIC), ΔAIC relative to the best-supported model, root mean square error (RMSE), and the squared correlation (R²) are provided for each model to illustrate relative performance. The logistic model provided the best fit, with the lowest AIC and RMSE and highest R².


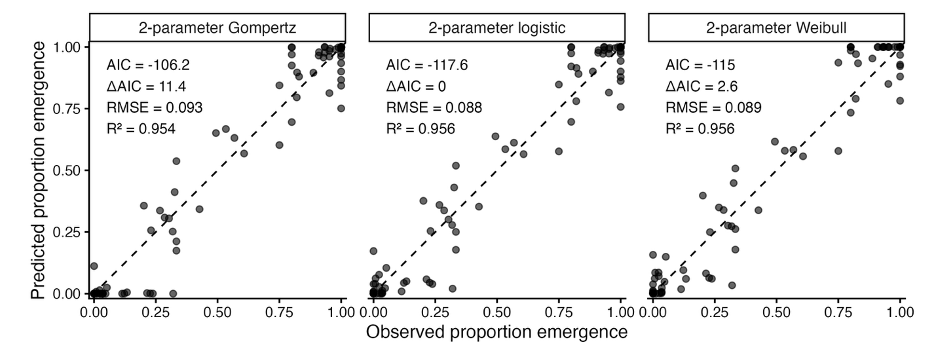

Supplement: nvag069_Supplementary_Data [file nvag069_supplementary_data.zip › Supplementary Figure S1.docx]
