# Supplementary material for: Seasonal phenology of Amauromyza karli (Diptera: Agromyzidae) in quinoa in San Luis Valley of Colorado
Source: Environ Entomol. 2026 Jun 23;55(3):nvag069. doi: 10.1093/ee/nvag069 (PMC13287995; doi:10.1093/ee/nvag069)
Supplement: nvag069_Supplementary_Data [file nvag069_supplementary_data.zip › Supplementary Table S1.docx]

Supplementary Table S1: Geographic locations and sampling periods for *Amauromyza karli* monitoring in quinoa grower fields in Colorado.

| Site code | Sampling Year | Coordinates (°N, °W) | County | Date of first trap placement | Date of last trap placement | Number of traps placed |
| --- | --- | --- | --- | --- | --- | --- |
| RG1 | 2022 | 37°42′34.8″N, 106°07′59.8″W | Rio Grande | 20 June | 10 Aug | 8 |
| AL1 | 2022 | 37°37′42.1″N,105°53′30.3″W | Alamosa | 27 June | 20 July | 8 |
| AL2 | 2022 | 37°38'17.5"N, 105°55'19.2"W | Alamosa | 6 June | 18 July | 8 |
| YU | 2022 | 40°16'11.6"N, 102°46'24.9"W | Yuma | 15 June | 10 July | 8 |
| RG1 | 2023 | 37°42′34.8″N, 106°07′59.8″W | Rio Grande | 24 May | 14 Aug | 6 |
| AL1 | 2023 | 37°37′42.1″N,105°53′30.3″W | Alamosa | 6 June | 14 Aug | 12 |
| YU1 | 2023 | 40°16'11.6"N, 102°46'24.9"W | Yuma | 13 June | 14 Aug | 6 |
| RG2 | 2024 | 37°42′45.9″N, 106°08′05.3″W | Rio Grande | 11 June | 13 Aug | 5 |
| AL1 | 2024 | 37°37′42.1″N,105°53′30.3″W | Alamosa | 28 April | 13 Aug | 5 |
| RG3 | 2024 | 37°42'28.3"N, 106°06'34.9"W | Rio Grande | 28 April | 13 Aug | 5 |
| AL1 | 2025 | 37°37′42.1″N,105°53′30.3″W | Alamosa | 10 June | 23 July | 5 |
| RG3 | 2025 | 37°42'28.3"N, 106°06'34.9"W | Rio Grande | 10 June | 23 July | 5 |
